# Circular RNA Eps15-homology domain containing protein 2 motivates proliferation, glycolysis but refrains autophagy in non-small cell lung cancer via crosstalk with microRNA-3186-3p and forkhead box K1

**DOI:** 10.1080/21655979.2022.2031385

**Published:** 2022-02-27

**Authors:** Fan Zhang, Tieying Zhang, ZiRan Zhao, Ying Ji, Yue Peng, Liang Zhao

**Affiliations:** a Department of Thoracic Surgery, National Cancer Center/National Clinical Research Center for Cancer/Cancer Hospital, Chinese Academy of Medical Sciences and Peking Union Medical College, Beijing, People’s Republic of China; b Department of Medical Oncology, JiLin Province People’s Hospital, Changchun City, JiLin Province, 130021, China

**Keywords:** Non-small cell lung cancer, circular RNA Eps15-homology domain containing protein 2, autophagy, glycolysis

## Abstract

Numerous studies have clarified the involvement of circular RNAs (circRNAs) in modulating malignant behavior of non-small cell lung cancer (NSCLC), while the concrete mechanism is not completely elucidated. The aim of the study was to figure out the latent functions and molecular mechanisms of circRNA Eps15-homology domain containing protein 2 (EHD2) on NSCLC proliferation, glycolysis and autophagy. The results clarified in NSCLC elevated expression of circEHD2 and declined expression of microRNA (miR)-3186-3p. Repressive circEHD2 or enhancive miR-3186-3p facilitated cell apoptosis rate and autophagy substrates LC3BII and Beclin-1, but curbed the colony-formation and DNA replication ability of NSCLC, glucose consumption, lactic acid production, glycolytic rate-limiting enzyme HK-2 and glutamine hydrolase GLS1 and P62, while overexpressed circEHD2 was adverse. Meanwhile, the impacts of repressive and elevated circEHD2 on NSCLC were turned around via reduced miR-3186-3p or forkhead box k1 (FOXK1) separately. Mechanically, FOXK1 was augmented via circEHD2ʹs competitive integration of miR-3186-3p. Depressive circEHD2 refrained NSCLC tumor growth, which was accelerated via enhancive one. All in all, circEHD2 accelerates the proliferation and glycolysis of NSCLC, but refrains autophagy and apoptosis via strengthening FOXK1 via the adsorption of miR-3186-3p, which is supposed to be a latent molecular target for NSCLC therapy later.

## Graphical abstract

**Figure:**
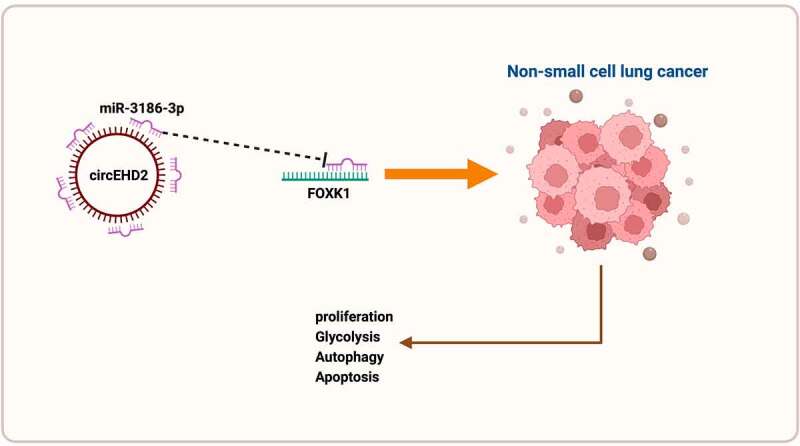

## 1. Introduction

Lung cancer (LC) is one of the tumors with high morbidity and mortality worldwide, among which the infected people of non-small cell LC (NSCLC) take up about 85% of LCs [1,2]. Despite certain advancement in NSCLC-linked therapy, the 5-year survival rate of NSCLC is still inferior to 15% [3,4]. Therefore, it is vital to study its molecular mechanism and explore brand-new therapeutic targets for NSCLC cure.

Circular RNA (circRNA) is a novel category of non-coding RNA with a sealed-loop structure. It is also a single-stranded RNA with covalently sealed 3 ‘and 5’ ends, lots of which are believed to participate in human diseases as gene modulators, which is usually achieved via interaction with other RNAs [5]. Recently, a number of studies have revealed the biological role and potential molecular mechanism of circRNA in NSCLC [6,7]. For instance, has Circ 0004050 memorably elevates DUSP9 protein expression in A549 cells via targeting miR-1233-3p, consequently repressing the ERK/JNK pathway. Meanwhile, hsa_Circ 0004050 is supposed to be a latent therapeutic target for NSCLC or a biomarker for later diagnosis [8]. Enhancive expression of circ-MTHFD2 has certain clinical values for the diagnosis, the pathological staging and prognosis of NSCLC [9]. A recent study affirms elevated expression of circEHD2 in renal cell carcinoma patients [10]. Nevertheless, the function of circEHD2 in NSCLC is ambiguous.

In this study, a comprehensive analysis of the potential molecular mechanism of circEHD2 influencing NSCLC advancement is manifested via clinical samples, *in vitro* and *in vivo* experiments. The results clarify that circEHD2 is available to motivate the proliferation and glycolysis, but repress autophagy and apoptosis of NSCLC via competitively binding to miR-3186-3p to mediate FOXK1 expression. This offers a brand-new target for later therapy or prevention of NSCLC.

## 2. Materials and methods

### 2.1. Clinical samples

From March 2016 to October 2018, 42 pairs NSCLC tissue and paired adjacent normal lung tissue were gained from NSCLC patients undergoing surgical resection in Cancer Hospital, Chinese Academy of Medical Sciences. The clinical diagnosis of NSCLC patients was confirmed via two pathologists making their own judgments independently. Chemotherapy or radiotherapy in the included patients was not received before specimen collection. Informed consent was signed by all participants prior to sample collection. After collection, the samples were quickly frozen in liquid nitrogen and then transferred at −80°C for subsequent studies. This study was approved via the Ethics Committee of Cancer Hospital, Chinese Academy of Medical Sciences and conducted in line with the *Declaration of Helsinki*.

### 2.2. Cell culture

Immortalized human bronchial epithelial BEAS-2B and NSCLC cells (A549, H1975, SK-MES-1 and H520) were gained from Shanghai Institute of Biochemistry and Cell Biology (Chinese Academy of Sciences, Shanghai, China). NSCLC cells were cultured in Roswell Park Memorial Institute 1640 medium (Thermo Fisher Scientific, Waltham, MA, USA) comprising 10% fetal bovine serum, 100 U/mL penicillin and 100 μg/mL streptomycin. BEAS-2B cells were maintained in bronchial epithelial basal medium (Lonza, Walkersville, Maryland, USA). All cells were cultured in an incubator containing 5% CO_2_ at 37°C in a humid environment. The cells were negative for mycoplasma contamination, and routinely passaged at 80% confluence.

### 2.3. Cell transfection

Small interfering RNA (siRNA) targeting circEHD2 (si-circEHD2) and FOXK1 (si-FOXK1) and negative control (si-NC) as well as overexpressed plasmids targeting circEHD2 (oe-circEHD2) and FOXK1 (oe-FOXK1) and NC (oe-NC) were designed and synthesized via GEkai Technology (Xuhui, Shanghai, China). MiR-3186-3p mimic/inhibitor and NC were purchased from Gemma (Shanghai, China). On the grounds of the kit protocol, the above reagents were transfected into H520 cells applying Lipofectamine 3000 (Fujun, Haidian, Beijing, China). Forty-eight hours after transfection, the transfection efficiency was detected by RT-qPCR, and the cells were collected for subsequent experiments.

### 2.4. Edu analysis

On the grounds of the kit protocol, EdU detection was conducted via using the Cell-light EdU Apollo 567 in vitro imaging kit (Ribobio Technology, Guangzhou, China) [11]. A fluorescence microscope (Nikon, Japan) was applied for measuring the percentage of EdU positive cells in five casual fields of each well. The experiment was repeated in triplicate (N = 3).

### 2.5. Colony formation assay

Colony formation assay was performed as described earlier [12]. The transfected H520 cells were seeded into 6-well plates (800 cells per well) and cultured for another 14 days. In the process of culture, the medium was updated twice each week. The cells were washed with phosphate buffer saline, fixed with methanol and stained with crystal violet for 20 min. Imaging was conducted via a Leica DMIL inverted light microscope (Leica Microsystems, Germany) and colonies in more than visible 50 cells were counted.

### 2.6. Flow cytometry

Apoptosis was detected as previously described [13]. Apoptosis was detected via the Annexin V-fluorescein isothiocyanate (FITC)/Propidium iodide (PI) apoptosis detection kit (KeyGen Biotech, Nanjing, China). H520 cells were reacted with 500 µL binding buffer and then treated with 5 µL Annexin V-FITC and 5 µL PI in the dark for 15 min. Finally, Attune NxT flow cytometry (Invitrogen) was applied for detecting apoptotic cells.

### 2.7. Glycolysis test

Glycolysis was studied in line with glucose consumption and lactic acid production, which were measured via a protocol of glucose assay kit (Biovision; Milpitas, CA, USA) and lactate assay kit (Biovision).

### 2.8. Reverse transcription quantitative polymerase chain reaction (RT-qPCR)

Gene expression was assessed as described earlier [14]. Total RNA was extracted from tissue specimens and cells via TRIzol reagent (Invitrogen). For complementary DNA (cDNA) synthesis of circEHD2 and FOXK1, RevertAid First Strand cDNA Synthesis Kit (Thermo Fisher Scientific) was applied for reverse transcription of 1 μg total RNA. For miR-3186-3p, cDNA was generated from total RNA via the TaqMan MicroRNA reverse transcription kit (Applied Biosystems, Foster City, CA, USA). Quantitative PCR amplification was conducted on LightCycler 480 Instrument II (Roche) via LightCycler 480 SYBR Green I Master Mix (Roche, Mannheim, Germany). Relative gene expression was evaluated via 2^−ΔΔCT^. Glyceraldehyde-3-phosphate dehydrogenase (GAPDH) was employed as an endogenous control for standardizing against circEHD2 and FOXK1, with U6 as a loading control for miR-3186-3p. Primer sequences were manifested below: circEHD2: forward: 5’-CTGGTGCGAGCTACGACTTC-3’, reverse: 5’-TCGTCCGAGATCTCCAGCTT-3’; miR-3186-3p: forward: 5’-GCGGCGGTCAAGAGCAATAACG-3’, reverse: 5’- ATCCAGTGCAGGGTCCGAGG-3’; FOXK1: forward: 5’-ACACGTCTGGAGGAGACAGC-3’, reverse: 5’- GAGAGGTTGTGCCGGATAGA-3’.

### 2.9. Western blot

Radio-Immunoprecipitation assay Lysis Buffer (Beyotime, Shanghai, China) was applied for extraction of total proteins from cells. Bicinchoninic acid Protein assay kit was used for assessment of protein concentration. The extracted proteins were separated via 10% sulfate polyacrylamide gel electrophoresis and electroblotted onto polyvinylidene fluoride membrane (Bio-Rad, CA, USA). Behind seal with 5% skim milk powder for 1 h, the membrane was incubated with a specific primary antibody overnight, and horseradish peroxidase conjugated secondary antibody at room temperature for 2 h. Western blots were examined via enhanced chemiluminescence (Rockford, USA) detection systems. Images were captured via the ImageQuant Las 4000 system (GE Healthcare, Chicago, IL, USA), and quantification of strip strength was via ImageQuant TL software (GE Healthcare). The applied primary antibodies were manifested as follows: FOXK1 (ab18196), p62 (ab56416), Beclin-1 (ab62557), GAPDH (ab8245) (all Abcam), LC3B (2775, Cell Signaling Technology).

### 2.10. The luciferase activity assay

Wild-type (WT) sequence of circEHD2 or FOXK1-3ʹuntranslated region (UTR) carrying miR-3186-3p binding sites was subcloned into pmirGLO vectors (Promega, Madison, WI, USA) with gaining circEDH2/FOXK1-WT reporter genes. Mutants of circEHD2 or FOXK1-3ʹUTR (circEDH2/FOXK1-MUT) were gained via applying the QuikChange XL Site-directed Mutagenesis Kit (Agilent Technologies, Santa Clara, CA, USA). The constructed luciferase reporter genes were transfected into H520 cells with miR-3186-3p mimic or its NC. After 48 h, the luciferase activity was detected via the dual luciferase reporter gene assay system (Promega). The relative luciferase activity was determined via normalizing the firefly against the renilla luciferase activities.

### 2.11. In vivo xenograft

All animal operations were carried out in strict accordance with the guidelines for the Care and Use of Experimental Animals of the National Institutes of Health of the United States, and authorized via the Animal Care and Use Committee of Cancer Hospital, Chinese Academy of Medical Sciences. Male BALB/c nude mice (6-week-old, 20–24 g) were purchased from SLAC Laboratory Animal Center (Shanghai, China) and adapted to the environment (at (24 ± 2)°C, 50%–60% humidity) before the experiment at least for 1 week. The mice were kept in a specific pathogen-free animal facility with randomly feeding standard rodent food and water. H520 cells (6 × 10^6^) transfected with oe/si-circEHD2 and the corresponding NC were subcutaneously injected into the left axilla of mice. Tumor size was measured via a vernier caliper and recorded every 7 d. The tumor volume was calculated via formula V = (length × width^2^ × 0.5). At 28 d after inoculation, the mice were euthanized and removal of tumor masses was conducted for subsequent determination.

### 2.12. Statistical analysis

SPSS 21.0 (SPSS Inc., Chicago, IL, USA) and GraphPad Prism 8.0 software were applied for statistical calculations and graphing, separately, with Student’s t-test for comparison of differences of the two groups. All data were clarified as mean ± standard deviation (SD) from at least three independent trials. *P* < 0.05 was accepted as indicative of distinct differences.

## 3. Results

### 3.1. CircEHD2 is highly expressed in NSCLC and implicated in clinical features

**Figure 1.**
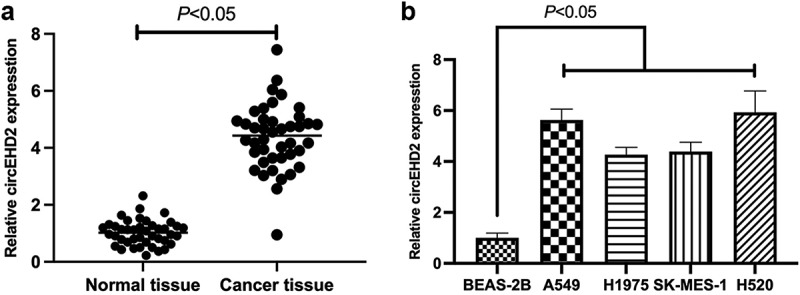
Elevated expression of circEHD2 is in NSCLC.

For exploration of the function of circEHD2 in NSCLC, circEHD2 expression was first examined in NSCLC tissues and cell lines via RT-qPCR, clarifying that the expression of circEHD2 in NSCLC tissues and cell lines was memorably higher versus para-cancer normal tissues and human bronchial epithelial cell line BEAS-2B (Figure 1(a,b)). This affirmed that circEHD2 was highly expressed in NSCLC and supposed to participate in NSCLC advancement.

(a/b). RT-qPCR to detect circEHD2 expression in NSCLC tissue and adjacent normal tissue, and human bronchial epithelial cell lines (BEAS-2B) and NSCLC cell lines (A549, H1975, SK-MES-1 and H520). Manifestation of the data was as mean ± SD (n = 3); *P < 0.05.

### 3.2. Silence or enhancing of circEHD2 expression represses and motivates NSCLC separately

**Figure 2.**
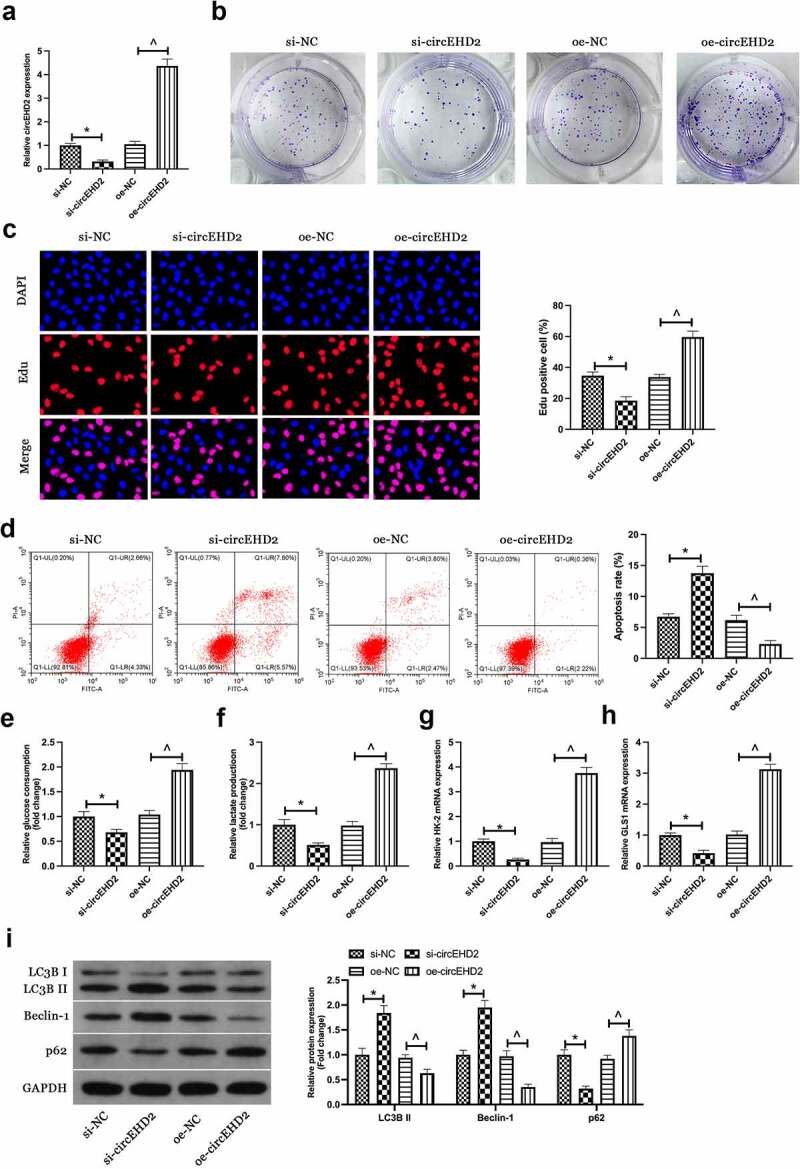
Silenced or overexpressed circEHD2 represses or motivates NSCLC separately.

For exploration of the biological function of circEHD2 in NSCLC, silence or enhancing of circEHD2 expression in H520 cells was conducted via transfection of si/oe-circEHD2 (Figure 2(a)). Subsequently, cell proliferation was examined by Edu analysis and colony formation assay. As shown in Figure 2(b,c). Silencing circEHD2 clearly inhibited proliferation and DNA replication of H520 cells, while overexpression of circEHD2 promoted them. Flow cytometry results showed that silencing circEHD2 facilitated apoptosis of H520 cells while overexpression of circEHD2 restrained it (Figure 2(d)). Subsequently, the effect of circEHD2 on glycolysis of cells was examined. The results manifested that silencing circEHD2 refrained glucose consumption and lactate production of H520 cells (Figure 2(e-f)), as well as the mRNA expression of glycolysis rate-limiting enzyme HK-2 and glutamine hydrolase GLS1 (Figure 2(g-h)), while overexpression of circEHD2 accelerated glycolysis in H520 cells. Subsequently, cell autophagy was examined by Western blot, as shown in Figure 2(i), silencing circEHD2 promoted the expression of LC3B II and Beclin-1, but inhibited the expression of p62, while overexpressed circEHD2 had the opposite effect. Briefly, these data clarified circEHD2 participated in the modulation of NSCLC proliferation, apoptosis, glycolysis, and autophagy.

(a). RT-qPCR to detect circEHD2 expression; (b). Colony formation assay to detect cell proliferation; (c). Edu assay to detect DNA replication; (d). The apoptosis rate determined via flow cytometry; (e, f). Glucose consumption and lactic acid production; (g, h). RT-qPCR to detect HK-2 and GLS1 mRNA expression; (i). LCB3, Beclin-1 and p62 protein expression detected via Western blot. In H520 cells transfected with si/oe-circEHD2. Manifestation of the data was as mean ± SD (n = 3); *P < 0.05.

### 3.3. MiR-3186-3p curbs NSCLC proliferation and glycolysis but expedites autophagy

**Figure 3.**
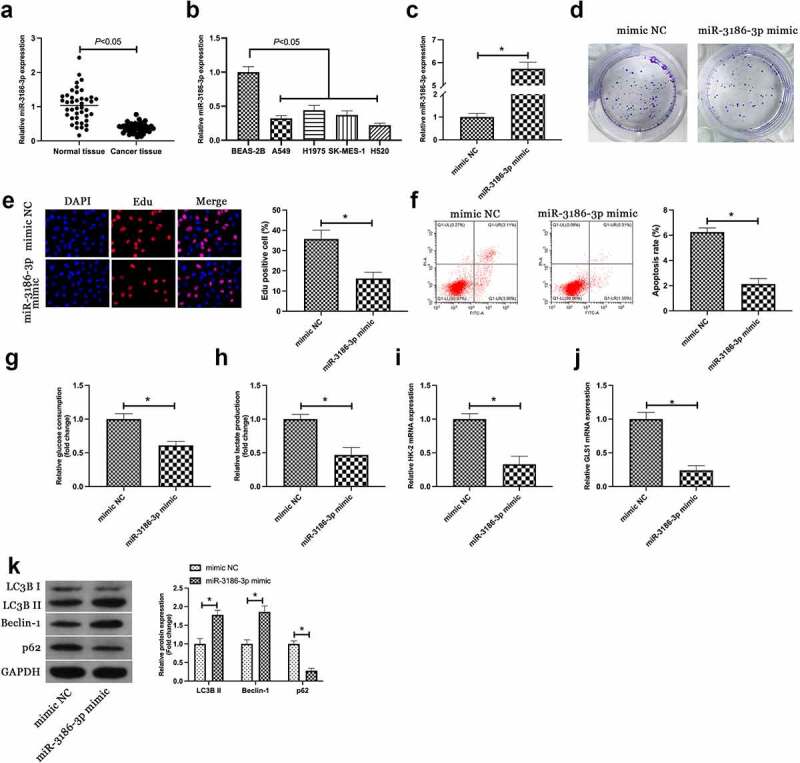
MiR-3186-3p curbs NSCLC proliferation, glycolysis and autophagy.

Next, the downstream miRNA modulated via circEHD2 was explored. A previous study has clarified the differential expression of miR-3186-3p in lung adenocarcinoma and miR-3186-3p can repress proliferation, migration but accelerate apoptosis of lung adenocarcinoma [15]. It was speculated a similar effect of miR-3186-3p in NSCLC. Firstly, miR-3186-3p expression was examined in NSCLC, and it came out the expression of miR-3186-3p in NSCLC patients and cell lines was higher versus normal tissues and BEAS-2B cells (Figure 3(a,b)). Subsequently, miR-3186-3p in H520 was upregulated via transfection with miR-3186-3p mimic (Figure 3(c)). Colony formation and Edu assays showed that up-regulation of miR-3186-3p inhibited proliferation and DNA replication of H520 cells (Figure 3(d,e)). Flow cytometry results manifested that up-regulation of miR-3186-3p promoted the proliferation of H520 cells (figure 3(f)). In addition, up-regulated miR-3186-3p also reduced glucose consumption and lactic acid production of H520 cells, and inhibited the mRNA expressions of HK-2 and GLS1 (Figure 3(g-j)). Western blot results showed that up-regulation of miR-3186-3p accelerated the protein expression of LC3B II and Beclin-1, but restrained the protein expression of p62 (Figure 3(k)). Briefly, the data clarified that miR-3186-3p was available to curb the malignant phenotype of NSCLC.

(a/b). RT-qPCR to detect miR-3186-3p expression in NSCLC tissue and adjacent normal tissue, BEAS-2B and NSCLC cell lines; (c). MiR-3186-3p detected via RT-qPCR; (d). Colony formation assay to detect cell proliferation; (e). Edu assay to detect DNA replication; F. The apoptosis rate determined via flow cytometry; (g, h). Glucose consumption and lactic acid production; I, J. RT-qPCR to detect HK-2 and GLS1 mRNA expression; K. Western blot to detect LCB3, Beclin-1 and p62 protein expression. C-K, in H520 cells introduced with miR-3186-3p mimic. Manifestation of the data was as mean ± SD (n = 3); *P < 0.05.

### 3.4. CircEHD2 competitively binds to miR-3186-3p which targets FOXK1

**Figure 4.**
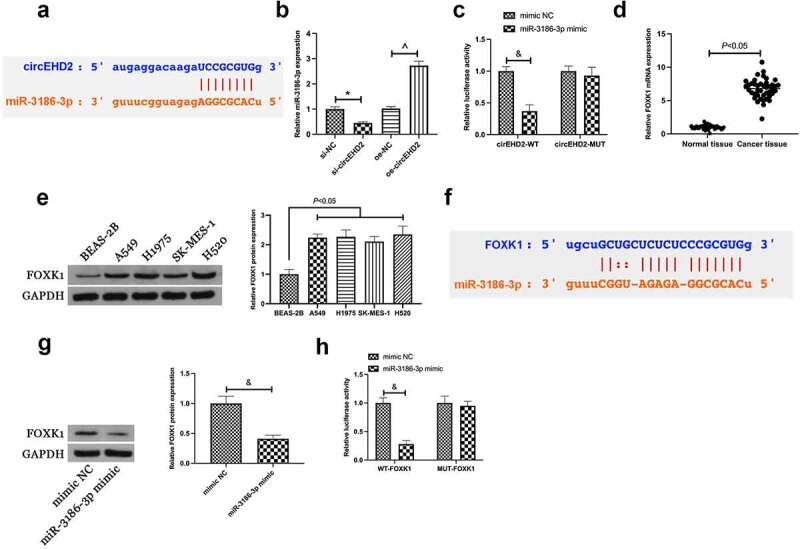
CircEHD2 competitively combines with miR-3186-3p which targets FOXK1.

Next, whether circEHD2 could be employed as a competing endogenous RNA for miR-3186-3p was explored. Via bioinformatics website http://starbase.sysu.edu.cn/ was discovered the latent binding sites of circEHD2 with miR-3186-3p (Figure 4(a)). In the meantime, it was affirmed elevation or reduction of miR-3186-3p expression in H520 cells after silencing or overexpression of circEHD2 (Figure 4(b)). Therefore, it was speculated that circEHD2 might take on a targeted binding link with miR-3186-3p. For verification of this hypothesis, a dual luciferase reporting assay was carried out, manifesting the obviously reduced luciferase activity after co-transfection of circEHD2-WT with miR-3186-3p mimic (Figure 4(c)). This manifested that circEHD2 competitively bound to miR-3186-3p.

(a). Bioinformatics website http://starbase.sysu.edu.cn/ to query circEHD2 and miR-3186-3p’s latent binding sites; B. RT-qPCR to examine miR-3186-3p expression in H520 cells transfected with si/oe-circEHD2; C. The luciferase activity assay verification of the targeting link of circEHD2 with miR-3186-3p; D. RT-qPCR to examine FOXK1 mRNA expression in NSCLC tissue and normal adjacent tissue; E. Western blot analysis of FOXK1 protein expression in BEAS-2B and NSCLC cell lines; F. Bioinformatics website http://starbase.sysu.edu.cn/ to query miR-3186-3p and FOXK1ʹs targeting link; G. Western blot detection of FOXK1 expression in H520 cells transfected with miR-3186-3p mimic; H. The luciferase activity assay verification of the targeting link of miR-3186-3p with FOXK1; Manifestation of the data was as mean ± SD (n = 3); *P < 0.05.

Subsequently, the downstream target gene of miR-3186-3p was explored. A former study clarifies the high expression of FOXK1 in NSCLC, and FOXK1 is available to expedite tumor growth *in vivo* [16]. In this study, it was also found the high expression of FOXK1 in NSCLC (Figure 4(d,e)). Through bioinformatics website http://starbase.sysu.edu.cn/ was discovered the latent binding sites of FOXK1 with miR-3186-3p (figure 4(f)). Hence, it was speculated that FOXK1 was supposed to be the target gene of miR-3186-3p. Meanwhile, it was discovered that overexpressed miR-3186-3p repressed FOXK1 expression in H520 cells (Figure 4(g)). Subsequently, a dual luciferase assay was performed to verify the targeting relationship, and the results clarified the reduced luciferase activity after co-transfection of FOXK1-WT and miR-3186-3p mimic (Figure 4(h)). Briefly, it was indicated that FOXK1 was the target gene of miR-3186-3p.

### 3.5. CircEHD2 influences NSCLC via modulating miR-3186-3p/FOXK1 pathway

**Figure 5.**
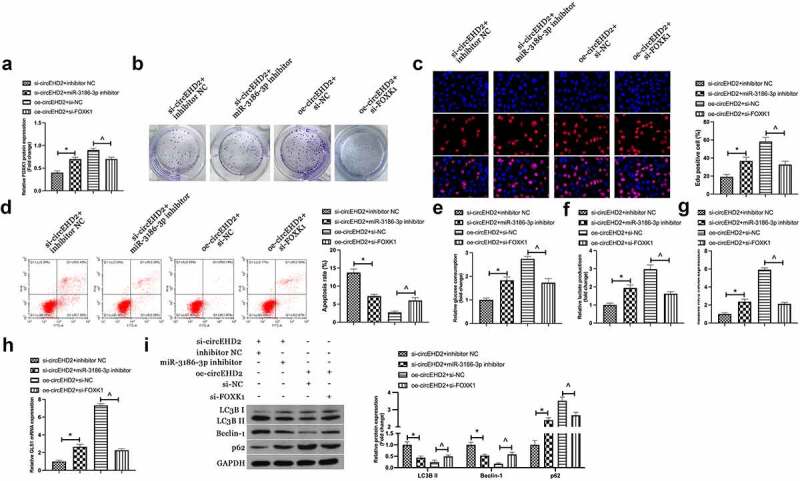
CircEHD2 impacts NSCLC via controlling miR-3186-3p/FOXK1 pathway.

Next, it was examined whether miR-3186-3p and FOXK1 took part in circEHD2ʹs influence of NSCLC. H520 cells were co-transfected with si/oe-circEHD2, miR-3186-3p inhibitor and si-FOXK1. Western blot results manifested that the repression and acceleration effects of FOXK1 protein expression via si/oe -circEHD2 were turned around via miR-3186-3p inhibitor and si-FOXK1 separately (Figure 5(a)). Additionally, functional assays showed that the refraining of si-circEHD2 on proliferation, DNA replication, glycolysis, and motivation of autophagy and apoptosis of H520 cells were restored via miR-3186-3p inhibitor. In contrast, the promoting effect of oe-circeHD2 on proliferation, DNA replication, glycolysis, and inhibition of apoptosis and autophagy of H520 cells were turned around via si-FOXK1 (Figure 5(b-i)).

(a). Western blot to detect FOXK1 protein expression; B. Colony formation assay to detect cell proliferation; C. Edu assay to detect DNA replication; D. The apoptosis rate determined via flow cytometry; E, F. Glucose consumption and lactic acid production; G, H. RT-qPCR to detect HK-2 and GLS1 mRNA expression; I. LCB3, Beclin-1 and p62 protein expression detected via Western blot. In H520 cells co-transfected with si-circEHD2 with miR-3186-3p inhibitor or /oe-circEHD2 and si-FOXK1. Manifestation of the data was as mean ± SD (n = 3); *P < 0.05.

### 3.6. *CircEHD2 expedites NSCLC tumor growth* in vivo

**Figure 6.**
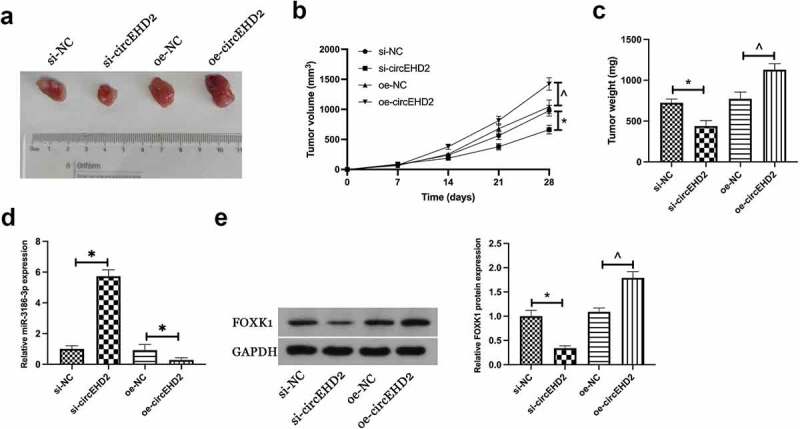
CircEHD2 motivates NSCLC tumor growth *in vivo.*

In order to further support the results of *in vitro* experiments, tumor xenotransplantation experiments were carried out subsequently. Results manifested that silencing circEHD2 reduced tumor volume, weight, and FOXK1 protein expression in NSCLC, but promoted miR-3186-3p expression, while overexpression circEHD2 had the opposite effect (Figure 6(a-e)). This suggested that circEHD2 promoted NSCLC tumor growth by regulating miR-3186-3p /FOXK1 axis.

(a). Representative images of tumor; B. Tumor volume; C. Tumor weight; D: MiR-3186-3p expression in tumor detected by RT-qPCR; E. FOXK1 protein expression in tumors detected via Western blot. Manifestation of the data was as mean ± SD (n = 6); *P < 0.05.

## 4. Discussion

NSCLC is a global public health problem and remains one of the most common cancers in the world [17]. The lack of effective interventions and specific molecular targets is the main cause of NSCLC treatment failure. Therefore, it is of great significance to find new molecular therapeutic targets for NSCLC. In this study, the function and action mechanism of circEHD2 in NSCLC were explored, manifesting that circEHD2 could motivate the proliferation, glycolysis but curb autophagy and apoptosis of NSCLC. Mechanically, the above impacts were achieved via circEHD2ʹs sponging on miR-3186-3p to mediate FOXK1.

Recently, circRNA has become a research focus in oncology. Accumulated studies manifest the tight link of circRNA with tumors. In LC, various circRNAs are confirmed to participate in proliferation, apoptosis, autophagy, invasion, and metastasis of NSCLC. For instance, circ_0010235 sponges miR-338-3p and plays a carcinogenic role in NSCLC cell proliferation, migration, and invasion via controlling KIF2A [18]. Yang B *et al*. announce that circ_0006677 refrains the progression and glycolysis of NSCLC via sponging miR-578 and controlling SOCS2 [19]. In this study, it was uncovered originally that circEHD2 served as a pro-oncogene in NSCLC to motivate proliferation, glycolysis, but repress autophagy.

Glycolysis and autophagy are crucial in the energy acquisition of cancer growth. When a tumor is out of control in division, its energy demands are tremendous. In the meantime, the glycolysis approach is frequently applicated during cancer cells’ gaining of energy even when oxygen is adequate [20]. Moreover, autophagy is a vital pathway for cancer cells to absorb glucose and amino acids from the tumor microenvironment [20]. While in early phases of cancer, augmented autophagy flux in tumor cells generally refrains the growth of tumor cells [21]. FOXK1, a tumor promoter gene, is highly expressed in all types of cancers, like NSCLC, breast cancer, liver cancer, and ovarian cancer, etc [22–24]. In this study, the results offered further data support for FOXK1 as a proto-oncogene. Additionally, a former study clarifies that FOXK1 is available to motivate chemoresistance via facilitating glycolysis in ovarian cancer [25]. A study also affirms that FOXK1 restrains autophagy in gastric cancer via mediating myc-associated zinc lipoprotein in acidic microenvironment [26]. In this study, it was discovered that FOXK1 silence was available to turn around the repressive effect of knockdown circEHD2 on glycolysis and motivation of autophagy in NSCLC, while augmented glycolysis and curbed autophagy offer the possibility of malignant metastasis of tumors.

Notably, a former study has testified that circEHD2 is identified as a diagnostic and prognostic biomarker in renal cell carcinoma [27]. Based on the results of this study, it was speculated that circEHD2 had a similar effect in NSCLC. Therefore, it was necessary to further determine the expression of circEHD2 in serum of NSCLC patients in subsequent studies, and determine whether circEHD2 could be used as a biomarker for early diagnosis of NSCLC through ROC curve analysis. In addition, it is necessary to determine the relationship between circEHD2 and survival prognosis in NSCLC patients in future studies, which will contribute to the diagnosis of poor prognosis in NSCLC patients. The limitation of this study is that there are few clinical data on circEHD2 in NSCLC, and it is not clear whether targeting circEHD2 has any effect on tumor improvement in NSCLC patients. In addition, the potential effect of circEHD2 on chemical resistance and distal metastasis of NSCLC needs to be determined in subsequent studies.

## 5. Conclusion

All in all, the results indicate circEHD2 sponges miR-3186-3p to mediate FOXK1, thereby motivating NSCLC proliferation and glycolysis, but repressing autophagy and apoptosis, clarifying that circEHD2 is able to be a latent therapeutic target for NSCLC.

## References

[1] Johnson AM, Hines RB, Johnson JA 3rd, et al. Treatment and survival disparities in lung cancer: the effect of social environment and place of residence. Lung Cancer. 2014;83:401–407. 24491311 10.1016/j.lungcan.2014.01.008

[2] Smith CB, Bonomi M, Packer S, et al. Disparities in lung cancer stage, treatment and survival among American Indians and Alaskan Natives. Lung Cancer. 2011;72:160–164. 20889227 10.1016/j.lungcan.2010.08.015

[3] Torre LA, Bray F, Siegel RL, et al. Global cancer statistics, 2012. CA Cancer J Clin. 2015;65:87–108. 25651787 10.3322/caac.21262

[4] Shi Y, Sun Y, Ding C, et al. [China experts consensus on icotinib for non-small cell lung cancer treatment(2016 version)]. Zhongguo Fei Ai Za Zhi. 2016;19:489–494. 27339727 10.3779/j.issn.1009-3419.2016.07.12 PMC5972963

[5] Chen LL, Yang L. Regulation of circRNA biogenesis. RNA Biol. 2015;12:381–388. 25746834 10.1080/15476286.2015.1020271 PMC4615371

[6] Zhang C-C, Li Y, Feng X-Z, et al. Circular RNA circ_0001287 inhibits the proliferation, metastasis, and radiosensitivity of non-small cell lung cancer cells by sponging microRNA miR-21 and up-regulating phosphatase and tensin homolog expression.[J]. Bioengineered. 2021;12:414–425. 33467964 10.1080/21655979.2021.1872191 PMC8806200

[7] Yunting Z, Shaolin Z, Tao W. Circular RNA hsa_circ_0002360 promotes non-small cell lung cancer progression through upregulating matrix metalloproteinase 16 and sponging multiple micorRNAs.[J], Bioengineered, 2021;undefined: undefined 10.1080/21655979.2021.1999370 PMC8809917 34747300

[8] Wang Y, Zang RK, Du YN. HSA_CIRC_0004050 on proliferation and apoptosis of A549 cells through ERK/JNK signaling pathway. J Biol Regul Homeost Agents. 2020;34:2037–2047. 33348975 10.23812/20-543-A

[9] Geng QQ, Wu QF, Zhang Y, et al. Clinical significance of circ-MTHFD2 in diagnosis, pathological staging and prognosis of NSCLC. Eur Rev Med Pharmacol Sci. 2020;24:9473–9479. 33015789 10.26355/eurrev_202009_23032

[10] Frey L, Klümper N, and Schmidt D, et al. CircEHD2, CircNETO2 and CircEGLN3 as diagnostic and prognostic biomarkers for patients with renal cell carcinoma. Cancers (Basel). 2021;13(9):2177. 33946584 10.3390/cancers13092177 PMC8124893

[11] Zhou J, Wang L, Sun Q, et al. Hsa_circ_0001666 suppresses the progression of colorectal cancer through the miR-576-5p/PCDH10 axis.[J]. Clin Transl Med. 2021;11:e565. 34841662 10.1002/ctm2.565 PMC8567033

[12] Zhijing R, Qinqin Y, Jiajia G, et al. Circular RNA hsa_circ_0000073 enhances osteosarcoma cells malignant behavior by sponging miR-1252-5p and modulating CCNE2 and MDM2.[J]. Front Cell Dev Biol. 2021;9:714601. 34568326 10.3389/fcell.2021.714601 PMC8459753

[13] Zhang D, Zhang G, Yu K, et al. Circ_0003204 knockdown protects endothelial cells against oxidized low-density lipoprotein-induced injuries by targeting the miR-491-5p-ICAM1 pathway.[J]. J Thromb Thrombolysis. 2021. undefined: undefined. 10.1007/s11239-021-02606-0 34797473

[14] Fan X, Yin X, Zhao Q, et al. Hsa_circRNA_0045861 promotes renal injury in ureteropelvic junction obstruction via the microRNA-181d-5p/sirtuin 1 signaling axis.[J]. Ann Transl Med. 2021;9:1571. 34790777 10.21037/atm-21-5060 PMC8576705

[15] Liu M, Wang P, Sui X, et al. Circular RNA circABCC4 regulates lung adenocarcinoma progression via miR-3186-3p/TNRC6B axis. J Cell Biochem. 2020;121:4226–4238. 31960988 10.1002/jcb.29627

[16] Niu J, Wang Y, Hu Y, et al. Mechanisms of miR-195-5p and FOXK1 in rat xenograft models of non-small cell lung cancer. Am J Transl Res. 2021;13:2528–2536. 34017411 PMC8129418

[17] Li B, Zhu L, Lu C, et al. circNDUFB2 inhibits non-small cell lung cancer progression via destabilizing IGF2BPs and activating anti-tumor immunity.[J]. Nat Commun. 2021;12:295. 33436560 10.1038/s41467-020-20527-z PMC7804955

[18] Zhu Y, Ma C, Lv A, et al. Circular RNA circ_0010235 sponges miR-338-3p to play oncogenic role in proliferation, migration and invasion of non-small-cell lung cancer cells through modulating KIF2A. Ann Med. 2021;53:693–706. 34024242 10.1080/07853890.2021.1925736 PMC8158223

[19] Yang B, Zhao F, Yao L, et al. CircRNA circ_0006677 inhibits the progression and glycolysis in non-small-cell lung cancer by sponging miR-578 and regulating SOCS2 expression. Front Pharmacol. 2021;12:657053. 34054537 10.3389/fphar.2021.657053 PMC8155686

[20] Amaravadi RK, Kimmelman AC, Debnath J. Targeting autophagy in cancer: recent advances and future directions. Cancer Discov. 2019;9:1167–1181. 31434711 10.1158/2159-8290.CD-19-0292 PMC7306856

[21] Ganapathy-Kanniappan S, Geschwind JF. Tumor glycolysis as a target for cancer therapy: progress and prospects. Mol Cancer. 2013;12:152. 24298908 10.1186/1476-4598-12-152 PMC4223729

[22] Liu Z, Chen P, Gao H, et al. Ubiquitylation of autophagy receptor optineurin by HACE1 activates selective autophagy for tumor suppression. Cancer Cell. 2014;26:106–120. 25026213 10.1016/j.ccr.2014.05.015 PMC4166492

[23] Zheng S, Yang L, Zou Y, et al. Long non-coding RNA HUMT hypomethylation promotes lymphangiogenesis and metastasis via activating FOXK1 transcription in triple-negative breast cancer. J Hematol Oncol. 2020;13:17. 32138762 10.1186/s13045-020-00852-y PMC7059688

[24] Cui H, Gao Q, Zhang L, et al. Knockdown of FOXK1 suppresses liver cancer cell viability by inhibiting glycolysis. Life Sci. 2018;213:66–73. 30312701 10.1016/j.lfs.2018.10.018

[25] Sun H, Wang H, Wang X, et al. Aurora-A/SOX8/FOXK1 signaling axis promotes chemoresistance via suppression of cell senescence and induction of glucose metabolism in ovarian cancer organoids and cells. Theranostics. 2020;10:6928–6945. 32550913 10.7150/thno.43811 PMC7295065

[26] Wang Y, Sun L, Qiu W, et al. Inhibiting forkhead box K1 induces autophagy to reverse epithelial-mesenchymal transition and metastasis in gastric cancer by regulating Myc-associated zinc finger protein in an acidic microenvironment. Aging (Albany NY). 2020;12:6129–6150. 32268297 10.18632/aging.103013 PMC7185099

[27] Frey L, Klümper N, Schmidt D, et al. CircEHD2, CircNETO2 and CircEGLN3 as diagnostic and prognostic biomarkers for patients with renal cell carcinoma.[J]. Cancers (Basel). 2021;13. undefined. 10.3390/cancers13092177 PMC8124893 33946584

